# A Lightweight Intrusion Detection System for Internet of Things: Clustering and Monte Carlo Cross-Entropy Approach

**DOI:** 10.3390/s25072235

**Published:** 2025-04-02

**Authors:** Abdulmohsen Almalawi

**Affiliations:** School of Computer Science & Information Technology, King Abdulaziz University, Jeddah 21589, Saudi Arabia; balmalowy@kau.edu.sa

**Keywords:** cybersecurity, machine learning (ML), classification, feature selection, IoT (Internet of Things), attacks, identification

## Abstract

Our modern lives are increasingly shaped by the Internet of Things (IoT), as IoT devices monitor and manage everything from our homes to our workplaces, becoming an essential part of health systems and daily infrastructure. However, this rapid growth in IoT has introduced significant security challenges, leading to increased vulnerability to cyber attacks. To address these challenges, machine learning-based intrusion detection systems (IDSs)—traditionally considered a primary line of defense—have been deployed to monitor and detect malicious activities in IoT networks. Despite this, these IDS solutions often struggle with the inherent resource constraints of IoT devices, including limited computational power and memory. To overcome these limitations, we propose an approach to enhance intrusion detection efficiency. First, we introduce a recursive clustering method for data condensation, integrating compactness and entropy-driven sampling to select a highly representative subset from the larger dataset. Second, we adopt a Monte Carlo Cross-Entropy approach combined with a stability metric of features to consistently select the most stable and relevant features, resulting in a lightweight, efficient, and high-accuracy IoT-based IDS. Evaluation of our proposed approach on three IoT datasets from real devices (N-BaIoT, Edge-IIoTset, CICIoT2023) demonstrates comparable classification accuracy while significantly reducing training and testing times by 45× and 15×, respectively, and lowering memory usage by 18×, compared to competitor approaches.

## 1. Introduction

**Figure 1:**
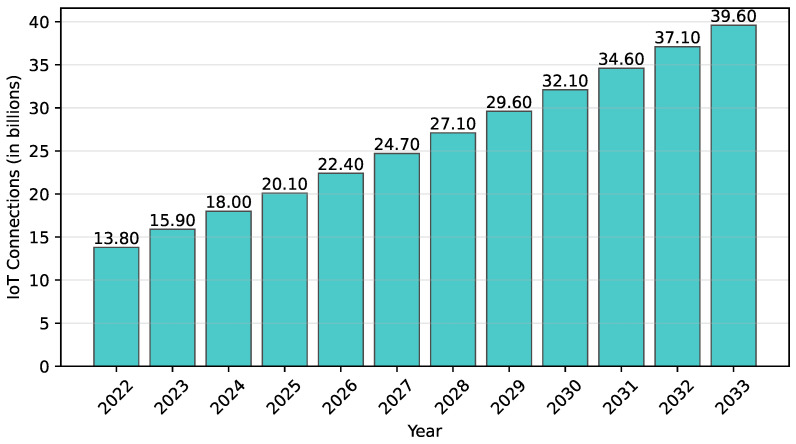
Number of IoT connections worldwide (2022–2023) with forecasts for 2024–2033 [2].

The Internet of Things (IoT) has become a transformative force in modern society, embedding smart objects into our daily lives and connecting them wirelessly through the Internet. These physical objects, equipped with electronics, software, and sensors, can communicate and exchange data, forming a network that has expanded rapidly. The economic impact of IoT is projected to grow from USD 3.9 trillion today to an estimated USD 11.1 trillion by 2025 [1]. A study on the IoT market reveals that the number of IoT connections worldwide increased from 13.8 billion in 2022 to 15.9 billion in 2023 [2], and this number is expected to grow significantly, reaching an estimated 39.6 billion connections by 2033, as illustrated in Figure 1. This growth encompasses a wide range of applications, from healthcare—where wearable devices monitor vital signs—to home automation, smart vehicles, and industrial automation in the Industrial Internet of Things (IIoT). The increasing reliance on IoT systems in both personal and industrial settings has significantly heightened concerns over security. As IoT systems are embedded with electronics and sensors connected via public networks, they are highly susceptible to a wide range of cyber threats, such as Denial-of-Service Attack (DoS) and injection attacks. In fact, cyber attacks have grown dramatically in recent years, rising by an astounding 776% in attacks between 100 Gbps and 400 Gbps from 2022 to 2023 [3]. These rapidly evolving threats highlights the urgent need for reinforced and resilient security measures to protect IoT systems from these evolving threats.

### 1.1. Motivation and Problem Statement

The increasing complexity of modern cyber threats highlights the need for more effective and adaptive security solutions, particularly in IoT environments. One of the most effective tools for combating cyber threats is the intrusion detection system (IDS) [4,5,6,7]. IDSs monitor network traffic for suspicious activities and alerts administrators when potential attacks are detected. Given the complexity of modern cyber threats, many researchers have turned to machine learning (ML) techniques to enhance IDS capabilities. ML techniques has been introduced as a valuable tool for developing IDSs, offering promising capabilities in detecting and classifying cyber attacks. ML techniques are particularly suited for identifying patterns in network traffic, enabling the detection of known attack behaviors and flagging anomalies. These systems can learn from large datasets and improve over time, making them well equipped to handle evolving threats. Despite these advancements, several fundamental challenges remain that must be addressed to develop more robust IDS solutions. ML-based IDSs require extensive and up-to-date datasets for effective training, which are often difficult to obtain [8]. Moreover, cyber attacks evolve rapidly, and by the time an IDS is retrained to include new attack signatures, additional threats may have already emerged. Another critical limitation is that traditional ML techniques face challenges in maintaining security due to the resource-constrained nature of IoT devices and the vast amount of data generated [9]. Thus, the limitations in handling the large, high-dimensional datasets typical of IoT environments must be addressed to ensure that IDSs can effectively detect threats without exhausting the limited computational resources and memory of IoT devices.

Effectively handling large-scale datasets and managing the resource constraints of IoT environments remains a critical challenge in designing efficient lightweight IDS solutions. Several approaches have been introduced to address the challenges associated with handling large-scale datasets and managing the inherent resource constraints of IoT environments. These methods mainly focus on optimizing data processing to ensure a balance between reducing the size of the training data and maintaining their diversity and representativeness, which is crucial, along with selecting relevant features that optimize detection accuracy [10,11,12]. Several sampling techniques, including random sampling and cluster-based sampling, have been proposed to tackle this issue to select representative observations for training, which ensures the behavioral diversity of the dataset is well captured. Sampling techniques [13,14], such as random sampling and cluster-based sampling, have been introduced to reduce data size. For instance, Meng et al. [13] proposed a random sampling technique based on interval timing for collecting wireless sensor network packets. While this method helps in reducing the volume of data used for training and decreases the computational load on IDSs, it has the limitation of potentially missing critical behaviors, leading to difficulties in detecting unobserved abnormal behaviors. Similarly, Hajj et al. [14] proposed a cluster-based sampling method using k-means to group the data generated by each IoT device into clusters. The centroid of each cluster is used to represent normal observations, while the most distant observation from the centroid within a normal cluster is flagged as abnormal. However, relying on centroids to represent normal and abnormal behaviors may fail to capture the full complexity and diversity of the data within the clusters. These limitations highlight the need for more advanced techniques that can more comprehensively represent the diversity of behaviors in IoT systems, ensuring that even subtle or rare patterns are not overlooked.

In addition to selecting representative representations, feature selection techniques have been widely used in IDSs for IoT systems, including wrapper-based [10,11] and filter-based methods [15,16,17]. Wrapper-based feature selection techniques have been widely used in IDSs for IoT systems because they evaluate subsets of features by training models and selecting those that optimize performance metrics, such as accuracy or F-score. However, the drawback of wrapper methods is their high computational cost, as they require training the model multiple times for different feature subsets, which reduces their effectiveness in settings with restricted resources. Filter-based methods have been proposed as an alternative to address this issue. Filter methods rank features based on statistical measures, such as correlation or relevance, without involving the learning algorithm, thus significantly reducing the computational burden. While filter techniques are more efficient, they come with their own limitations, such as not accounting for interactions between features, which can result in suboptimal performance when applied to more complex datasets. Though filter techniques are computationally feasible for IoT, they may sacrifice accuracy in some cases. Thus, achieving an effective balance between computational efficiency and detection accuracy remains an open challenge in feature selection for IDSs in IoT environments.

### 1.2. Our Contributions

The major contribution of this paper is addressing the limitations of feature selection techniques and the challenges in choosing the most representative observations to enhance the efficiency and effectiveness of IDSs in IoT environments. To achieve this, we introduce a novel two-fold approach for developing a lightweight IoT-based IDS approach, focusing on overcoming critical limitations in both feature selection and observation selection.

In the first step, we propose a recursive clustering method that integrates compactness and entropy-driven sampling to condense large datasets, ensuring that essential behavioral patterns are retained. Unlike traditional clustering techniques, which require a predefined number of clusters and are less adaptable to datasets with variable densities, our recursive clustering approach dynamically partitions data based on their inherent characteristics, eliminating the need for a predefined cluster count. This adaptability enables our method to capture diverse patterns of both normal and abnormal behavior, an essential requirement for effective intrusion detection in IoT systems. By measuring intra-cluster distances using compactness and evaluating diversity within each cluster through entropy, our method strikes a balance between reducing dataset size and preserving critical behavioral diversity. This approach ensures that condensed datasets retain essential characteristics, which is crucial for training models in resource-limited IoT environments.

For the second step, we introduce the Monte Carlo Cross-Entropy approach for feature selection, integrated with a stability metric of features to ensure that the most stable and relevant features are selected across multiple subsets of the dataset. Unlike traditional feature selection methods that are typically applied to the entire dataset—which is computationally intensive—our approach uses the Monte Carlo Cross-Entropy approach on smaller data subsets. This reduces computational overhead and enhances the scalability of the feature selection process, making it more suitable for large datasets commonly encountered in IoT environments. The inclusion of a stability metric adds robustness by ensuring that the selected features are not only relevant but also consistently chosen across different data subsets. This consistency helps prevent overfitting and ensures that the IDS maintains high detection accuracy across varying data distributions. Collectively, these contributions significantly advance the development of lightweight, scalable, and efficient IDS solutions for resource-constrained IoT systems, capable of maintaining performance even with large-scale, high-dimensional data. Below is the summary of the contributions of this paper.

- Recursive Clustering for Data Condensation: We propose a recursive clustering method that integrates compactness and entropy-driven sampling to select a small, highly representative set of observations from the larger training dataset, while preserving key behavioral patterns.
- Monte Carlo Cross-Entropy-Based Feature Selection: We adopt a Monte Carlo Cross-Entropy approach combined with a stability metric of features to consistently select the most stable and relevant features, enhancing the performance of a lightweight IoT-based IDS.
- Efficient IoT-based lightweight IDS: This approach utilizes data condensation and feature selection to reduce computational overhead while preserving or enhancing classification performance. Such efficiency is critical for IoT environments, where both computational resources and memory are often limited.
- Evaluation on Real-World IoT Datasets: We evaluate our proposed approach using IoT datasets from real devices (N-BaIoT [18], Edge-IIoTset [19], CICIoT2023 [20]), demonstrating that our condensed dataset achieves comparable accuracy while reducing training and testing times, and memory usage.

### 1.3. Structure

The rest of this paper is structured as follows: In Section 2, we review the literature on intrusion detection systems for IoT and feature selection techniques. Section 3 details our proposed two-fold approach in detail. Section 4 presents the evaluation of our approach using various IoT datasets and classifiers. Finally, Section 6 concludes this paper and highlights possible directions for future work. The list of acronyms used throughout this paper are listed in Table 1 to ensure clarity for the reader.

## 2. Related Work

The proposed approach is mainly related to the following four lines of research.

### 2.1. IoT Security Mechanisms

In recent years, IoT has been widely adopted in various fields. This technology has become an integral part of our daily lives, from monitoring the temperature in our homes to controlling lights, refrigerators, and other appliances. However, as these systems increasingly rely on public networks and standard protocols such as TCP/IP, they are becoming more vulnerable to cyber threats. These vulnerabilities expose IoT systems to a wide range of attacks, such as denial of service (DoS), distributed denial of service (DDoS), injection attacks, etc. [21,22,23]. However, traditional security mechanisms are not well suited to protect these systems due to their inherent characteristics, including limited computational power and resources.

In the literature, many security measures have been proposed to protect IoT devices, including cryptography [24,25], access control mechanisms [26,27], and IDSs [6,7]. IDSs are considered the first line of defense in cybersecurity due to their ability to monitor system behavior and detect potential security breaches in near real time. The daily data generated by IoT devices are impractical for humans to monitor and analyze manually. Consequently, ML techniques have emerged as a promising approach in developing IDS solutions for IoT, as it can learn and recognize normal behavior and detect deviations that may indicate malicious activity. To address the resource constraints inherent in the IoT, researchers continually strive to design IDSs that comply with these limitations. Given that large and high-dimensional datasets present significant challenges in the development of such systems, various IDSs have been proposed in the literature to address these challenges effectively. Some approaches focus on feature selection [10,11,12,28,29,30] and observation reduction [13,14] to optimize performance, while others explore decentralized learning techniques such as Federated Learning (FL), which not only improves privacy by keeping data localized but also facilitates intrusion detection in distributed IoT environments [31,32]. However, this work does not focus on FL but instead addresses IDS challenges through feature selection and observation reduction.

### 2.2. Feature Selection in IDS

Feature selection-based IDSs have been widely proposed in the literature to enhance detection accuracy and reduce computational costs. The open issues in feature selection techniques lie in the identification of a few and most relevant features that maximize detection accuracy. Wang et al. [10] adopted a wrapper-based approach to identify the most relevant features. Their method utilizes the F-score and accuracy metrics to evaluate candidate characteristics, ultimately selecting those that maximize both metrics.

Similarly, Shafiq et al. [11] used a wrapper-based method. In their approach, they introduced a mathematical technique called the Bijective Soft Set Method, which establishes a precise one-to-one mapping between features and their respective outcomes. This method serves as a filtering technique, applied prior to the wrapper-based approach, to eliminate irrelevant or redundant features early in the process. As in the previous study, their wrapper-based technique evaluates each candidate subset of features using the accuracy metric, ensuring the selection of an optimal feature set.

A wrapper-based approach was also proposed in [12], adapting the Gray Wolf Optimization (GWO) technique to identify the most relevant features that optimize the performance of an SVM classifier, thereby improving its ability to differentiate between normal and abnormal behavior. In [28], a cost-aware feature selection approach based on cross-entropy was introduced to optimize relevant features while considering computational, memory, and privacy costs. This method employs cross-entropy minimization to iteratively refine feature selection by evaluating candidate subsets and adjusting their probabilities. Unlike traditional feature selection techniques that focus solely on classification accuracy, this approach balances detection performance with resource efficiency, making it particularly suitable for deployment in constrained environments such as IoT and edge computing.

More recently, Li et al. [29] proposed a Black Hole Optimization (BHO) technique to identify the smallest subset of features that maintain high accuracy and minimize classification errors. The selected features are then used to train a Parallel Convolutional Neural Network (PCNN), which consists of two separate CNNs for handling different representations of the data.

In [30], a filter-based feature selection technique was proposed to introduce a lightweight and efficient IDS for IoT networks, emphasizing energy efficiency, detection accuracy, and computational constraints. This work combines a Stochastic Gradient Descent Classifier (SGDC) with a ridge regressor-based feature selection algorithm to optimize both classification performance and resource efficiency. Four ridge regression-based techniques were adopted: importance-coefficient, correlation-coefficient, forward-sequential, and backward-sequential selection. In the proposed approach, each selection criterion was individually evaluated to determine which method maximizes accuracy while reducing computational cost. However, despite their speed in learning relevant features, filter-based techniques typically exhibit lower accuracy compared to wrapper-based methods.

### 2.3. Observation Reduction in IDS

Despite the effectiveness of feature reduction techniques, several challenges persist in developing lightweight models, especially when dealing with large-scale training datasets. A critical additional challenge is determining how to effectively reduce the size of the training dataset itself without compromising its ability to accurately capture and represent the behavioral patterns of the system. Meng et al. [13] proposed a random sampling technique based on interval timing for collecting wireless sensor network packets. The primary objective of this sampling process is to reduce the volume of data used for training, thereby decreasing the computational load on the IDS. However, this technique may not capture all relevant behaviors, potentially leading to difficulties in detecting unlearned abnormal behaviors. Consequently, this approach contrasts with the proposed method, which focuses on learning representative observations from the entire dataset to ensure comprehensive detection capabilities. Similar to the first phase of the proposed approach, which focuses on learning representative training data, Hajj et al. [14] have proposed a cluster-based sampling method using k-means to group the data generated by each IoT device into several clusters. The centroid of each cluster is used as a representative of normal observations, while the first observation identified as abnormal, or the most distant observation from the centroid within a normal cluster, is flagged as abnormal. However, relying solely on centroids, which represent normal and abnormal behavior within each cluster, may not fully capture the diversity or complexity of the data within the clusters. In contrast, the proposed approach for learning representatives is based on recursive clustering, which does not require the number of clusters to be specified as a parameter that could influence the clustering outcome. This method prioritizes the representation of various types of behaviors, ensuring that even within a single type, variations are adequately captured and preserved.

As can be seen from the related work, the existing IDS in IoT environments suffers from two main issues. The first issue is the selection of representative observations to capture the full diversity of attack patterns. The second issue is selecting optimal and stable features to balance computational efficiency and detection accuracy. To address these challenges, we propose a novel two-fold approach that enhances both observation selection and feature selection for lightweight IDSs in IoT environments. In the first step, Recursive Clustering with Compactness and Entropy-Driven Sampling is introduced to learn the most representative observations through recursive clustering. This ensures essential behavioral diversity and effective attack detection while reducing dataset size. In the second step, Monte Carlo Cross-Entropy Feature Selection with Stability Metrics is proposed to select the most relevant and stable features that remain consistent across different observations. This improves the computational efficiency of IDS solutions in resource-constrained IoT environments and enhances the detection accuracy.

## 3. The Proposed Approach

**Figure 2:**
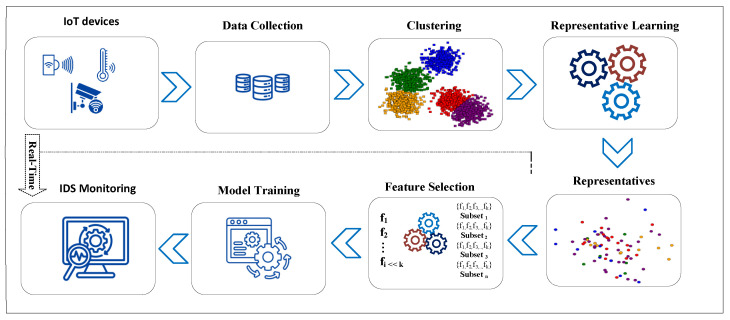
Overview of the enhanced intrusion detection system for Internet of Things.

In this section, we present our proposed approach for designing an efficient IoT-based IDS approach, considering the limited resources of IoT devices and focusing on developing lightweight detection models. To address this challenge, we propose a two-fold approach: selecting representative observations and identifying nearly optimal features. Specifically, the process of selecting representative observations includes multiple steps, such as employing a recursive clustering technique based on the *k*-means algorithm, combined with sampling and scoring methods. For selecting the most relevant features, we adopt the Monte Carlo Cross-Entropy optimization technique, coupled with a stability metric, to consistently identify and focus on the most stable features across different subsets of the dataset. The overall workflow of our approach is illustrated in Figure 2. In addition, Algorithm 1 outlines the general steps for selecting representative observations and identifying nearly optimal features.
**Algorithm 1:** The general steps for proposed approach

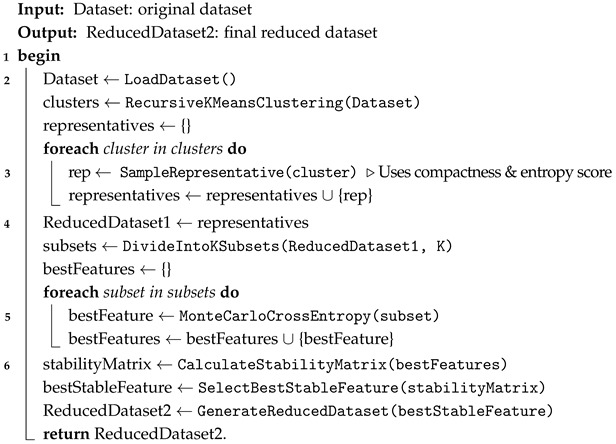


### 3.1. Representative Observations

In this section, we introduce the first fold of our proposed approach, which involves selecting the most representative observations that can represent and maintain the integrity of the original dataset. The resultant representative dataset, hereafter sometimes referred to as the condensed dataset, can help develop an efficient IoT-based lightweight IDS while maintaining or even improving the classification performance over using the original dataset for monitoring systems. Specifically, we propose a recursive clustering technique based on k-means combined with sampling and scoring methods, as discussed in the following subsections.

#### 3.1.1. Recursive Clustering

The *k*-means clustering algorithm [33] is widely used in data analysis and ML techniques to partition a dataset into *k* distinct clusters, where each cluster contains observations that are assumed to be similar to one another based on a distance metric, such as the Euclidean distance. In our proposed technique, we adapt the *k*-means algorithm to recursively partition the data into manageable clusters. We do not use *k*-means to discover inherent clusters; instead, we allow multiple clusters to share the same class label. Our goal is to group the most similar observations together to facilitate the selection of representative observations for each cluster or group. The proposed clustering technique is governed by a specified cluster size threshold, and any cluster that exceeds this threshold is recursively partitioned further.

**Figure 3:**
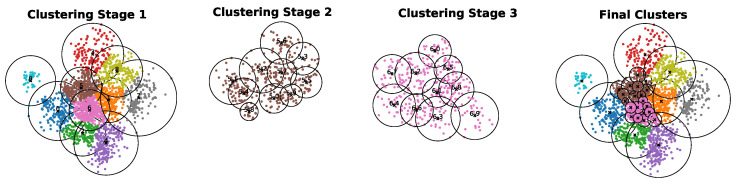
Overview of the recursive k-means algorithm for data partitioning.

Figure 3 illustrates the process of recursive clustering, which is shown in the sub-figures. The first sub-figure, Clustering Stage 1, displays the initial clusters after partitioning the data into 10 clusters. As observed, clusters 5 and 6 exceed the threshold of 250. Therefore, cluster 5 is recursively partitioned into 10 clusters, as depicted in Clustering Stage 2. Similarly, cluster 6 is recursively partitioned into 10 clusters, as shown in Clustering Stage 3. After this stage, all clusters are below or equal to the threshold. At this point, we stop the recursive clustering. This final stage demonstrates how the proposed recursive *k*-means algorithm partitions the data into groups or clusters containing the most similar observations.

To formalize the recursive *k*-means clustering process, let $X_{\mathrm{obs}}$ be a dataset with *M* observations and *N* features, denoted as follows:

$$X_{\mathrm{obs}}=\left(\begin{array}{cccc} X_{11} & X_{12} & \cdots & X_{1N}\\ X_{21} & X_{22} & \cdots & X_{2N}\\ \vdots & \vdots & \ddots & \vdots \\ X_{M1} & X_{M2} & \cdots & X_{MN} \end{array}\right)$$

where $X_{ij}$ represents the value of the *j*-th feature of the *i*-th observation. The Euclidean distance between two observations $x_{i}$ and $x_{j}$ is defined as follows:

(1) $$d\left(x_{i},x_{j}\right)=\sqrt{\sum_{k=1}^{N}{\left(x_{ik}-x_{jk}\right)}^{2}}$$

The *k*-means clustering algorithm partitions the dataset into *k* clusters by randomly selecting *k* observations and initially assuming them as the centroids of the clusters $\left\{c_{1},c_{2},\ldots ,c_{k}\right\}$. An observation $x_{j}$ is assigned to cluster $c_{i}$ if the distance between $x_{j}$ and $c_{i}$ is less than or equal to the distance between $x_{j}$ and any other cluster $c_{m}$, which is defined as $x_{j}\in c_{i}\;\mathrm{if}\;d\left(x_{j},c_{i}\right)\leq d\left(x_{j},c_{m}\right)\;\forall m\neq i$, where *m* represents any other cluster different from *i*. Afterward, the centroid of each cluster is updated as $c_{i}=\frac{1}{|c_{i}|}\sum_{x_{j}\in c_{i}}x_{j}$, where $|c_{i}|$ is the number of observations in cluster $c_{i}$. This process is repeated until the centroids converge. If the size of any resultant cluster $c_{i}$ exceeds a predefined threshold *T* (i.e., $|c_{i}|>T$), then $c_{i}$ is recursively partitioned into smaller clusters $c_{i1},c_{i2},\ldots ,c_{ik}=\mathrm{k-means}\left(c_{i},k\right)$. This recursive process continues until all cluster sizes are less than or equal to the threshold *T*. The steps of this recursive clustering process are summarized in Algorithm 2.

Following the recursive clustering process, we obtained several clusters, each of which is assumed to contain similar observations. Naturally, some clusters are tightly grouped, while others are more dispersed. Therefore, the number of representative observations selected from each cluster should be determined by its distribution characteristics. To address this, we propose a sampling technique that determines the number of representative observations for each cluster based on its compactness and spatial entropy.
**Algorithm 2:** Recursive k-means clustering
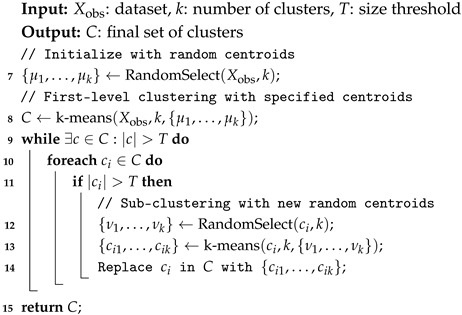


#### 3.1.2. Cluster Compactness

The compactness score of a cluster measures the average intra-cluster distance and indicates the cluster’s distribution [34]. Additionally, it provides insight into the cluster’s density and cohesion. A high compactness score indicates that observations within the cluster are closely grouped, suggesting that a small number of observations can effectively represent the cluster due to their high similarity. In other words, a small number of representative observations can capture the core characteristics of the cluster, regardless of its size. Given a set of clusters $C=\{c_{1},c_{2},\ldots ,c_{k}\}$ generated by Algorithm 2, the intra-compactness score (IC) for a cluster $c_{i}$ is calculated as follows:

(2) $$\mathrm{IC}\left(c_{i}\right)=\frac{2}{n(n-1)}\sum_{i=1}^{n-1}\sum_{j=i+1}^{n}d\left(x_{i},x_{j}\right)$$

Here, *n* represents the number of observations in cluster $c_{i}$, and $d(x_{i},x_{j})$ is the Euclidean distance as defined in Equation (1).

#### 3.1.3. Cluster Entropy

Entropy is a measure used in information retrieval and information theory to quantify uncertainty. A higher entropy value indicates greater disorder and reduced predictability, signifying increased uncertainty within the system [35]. In this paper, we propose the spatial entropy score for each cluster to measure variability and diversity within the cluster. This score also helps us estimate the number of representatives to select from each cluster. A high spatial entropy indicates a more scattered distribution, suggesting a need for more representative samples to capture the diversity of the cluster. Given a set of clusters $C=\{c_{1},c_{2},\ldots ,c_{k}\}$ generated by Algorithm 2, the spatial entropy score (SES) for a cluster $c_{i}$ is calculated by first identifying the closest neighbors for each observation within the cluster $c_{i}$ using the Euclidean distance, as defined in Equation (1). To proceed, we must first establish the definition of *k*-nearest neighbors for an observation *p*.

**Definition** **1**  **(K-Nearest Neighbors (KNNs)).** *Let $c_{i}$ be one of the clusters generated by Algorithm 2. The KNNs of an observation p in $c_{i}$ are the observations whose distances are the closest to the query observation p compared to the remaining observations in $c_{i}$. Let $X=\{x_{1},x_{2},\ldots ,x_{n}\}$ be a set of n observations in cluster $c_{i}$, and let $d(x_{i},x_{j})$ be the distance function between pairs of observations. Given $S=\{x_{1},x_{2},\ldots ,x_{k}\}$ as the set of KNNs of observation p, where S⊂X, p∈X, and p∉S, S is the KNNs of p if $\max_{n=k}d\left(p,S_{n}\right)<\min_{n\neq i}d\left(p,Z_{n}\right)$ holds, where Z=X∖(S∪{p}).*

After the KNNs of the observation *p* are determined as $S=\{x_{1},x_{2},\ldots ,x_{k}\}$, the distances are converted to weights using the exponential function to give higher importance to closer observations. This approach emphasizes local relationships while reducing the influence of distant observations [36]: $w_{ij}=\exp\left(-d\left(x_{i},x_{j}\right)\right),$ where $w_{ij}$ represents the weight assigned to the *j*-th nearest neighbor of observation $x_{i}$. Then, we normalize the weight $w_{ij}$ of the *j*-th nearest neighbor with respect to the sum of the weights of all KNNs of $x_{i}$ to ensure that the sum of the probabilities for the KNNs is 1. This normalization is essential for obtaining probabilities to calculate the local entropy of $x_{i}$. Formally, the calculation of normalization and entropy is defined as follows: $p_{ij}=\frac{w_{ij}}{\sum_{j=1}^{k}w_{ij}}$, where $\sum_{j=1}^{k}p_{ij}=1$. This is crucial for calculating the local entropy of the KNNs of $x_{i}$, which is defined as follows:

$$H\left(x_{i}\right)=-\sum_{j=1}^{k}p_{ij}\log p_{ij}$$

Then, we exponentially normalize the entropy score for each observation $x_{i}$, as defined below. This is to highlight the significant differences between these scores [36]:

$$H_{\exp}\left(x_{i}\right)=\frac{e^{H(x_{i})}}{\sum_{i=1}^{k}e^{H(x_{i})}}$$

After calculating the entropy scores for each observation in a cluster $c_{i}$, we compute the average entropy score for this cluster $c_{i}$ using the following formula, where *n* is the number of observations in cluster $c_{i}$:

(3) $$H\left(c_{i}\right)=\frac{1}{n}\sum_{i=1}^{n}H_{\exp}\left(x_{i}\right)$$

#### 3.1.4. Sample Size Determination

As previously discussed, the training data are partitioned into a number of clusters using a recursive scheme in clustering. Each cluster inherently possesses unique characteristics in terms of distribution, variability, and diversity. As discussed, we propose two metrics to choose representative data for each cluster, namely cluster compactness and entropy scores, which are defined in Equations (2) and (3), respectively. By leveraging these two measures, we can select the observations that best represent their respective clusters. Clusters with low compactness and high entropy require a larger sample size to capture their variability accurately. In contrast, clusters with high compactness and low entropy require fewer representatives due to their homogeneity. Therefore, we use the following formula to calculate the combined score for both compactness and entropy scores for a cluster $c_{i}$:

(4) $$\mu_{i}=\alpha \times IC\left(c_{i}\right)+\left(1-\alpha \right)\times H\left(c_{i}\right)$$

where $\mu_{i}$ is the resulting score after combining the two metrics. The weighting factor α (between 0 and 1) determines the relative importance of each metric, balancing the trade-off between compactness and entropy. Using $\mu_{i}$, the number of observations from each cluster is defined as follows:

(5) $$s_{i}=\mu_{i}\times \left|c_{i}\right|$$

In the process of selecting number of representative observations, we introduce λ to define the total desired number of observations for the training process. For instance, when λ is set to 1%, it indicates that the representative observations will account for 1% of the entire dataset. Therefore, we normalize the final number of representative observations for each cluster using the following normalization factor:

(6) $$N_{f}=\frac{\lambda \times N}{\sum_{i=1}^{k}s_{i}}$$

where *N* is the total number of observations in the dataset, and *k* is the number of generated clusters. The final size of the representative observations for each cluster after normalization is defined as follows:

(7) $$o_{i}=\max\left(1,\mathrm{round}(s_{i}\times N_{f})\right)$$

where $o_{i}$ represents the number of representative observations for cluster $c_{i}$. The new dataset, $X_{\mathrm{sub}}$, is then constructed based on the representative observations for each cluster as follows:

(8) $$X_{\mathrm{sub}}=\overset{k}{\underset{i=1}{⋃}}\mathrm{RandomSample}\left(c_{i},o_{i}\right)$$

In this formulation, $X_{\mathrm{sub}}$ represents the surrogate dataset, which is created by selecting $o_{i}$ representative observations from each cluster $c_{i}$ using a random sampling technique. The process of selecting representative observations is summarized in Algorithm 3.

##### An Illustrative Example

This example demonstrates how the size determination process is performed. Assume we have three clusters, $c_{1}$, $c_{2}$, and $c_{3}$, with sizes of 50, 30, and 20, respectively, and their compactness and entropy scores are (0.5, 0.7, 0.2) and (0.3, 0.4, 0.6), respectively. Using a weight parameter α set to 0.5, we calculate the combined scores for each cluster as defined in Equation (4), resulting in combined scores of 0.42 for $c_{1}$, 0.58 for $c_{2}$, and 0.36 for $c_{3}$. Based on these combined scores, the number of representative observations for clusters $c_{1}$, $c_{2}$, and $c_{3}$ are 21, 17.4, and 7.2, respectively, giving a total of 45.6 observations for the entire dataset. If we assume the total dataset size is 100 observations and λ is set to 2%, we calculate the normalization factor $N_{f}$ as the proportion of λ with respect to the dataset size, divided by the estimated total number of representative observations. This yields $N_{f}=\frac{0.02\times 100}{45.6}\approx 0.4386$. The final sizes of representative observations for clusters $c_{1}$, $c_{2}$, and $c_{3}$ are then calculated as 9, 8, and 3, respectively.

**Figure 4:**
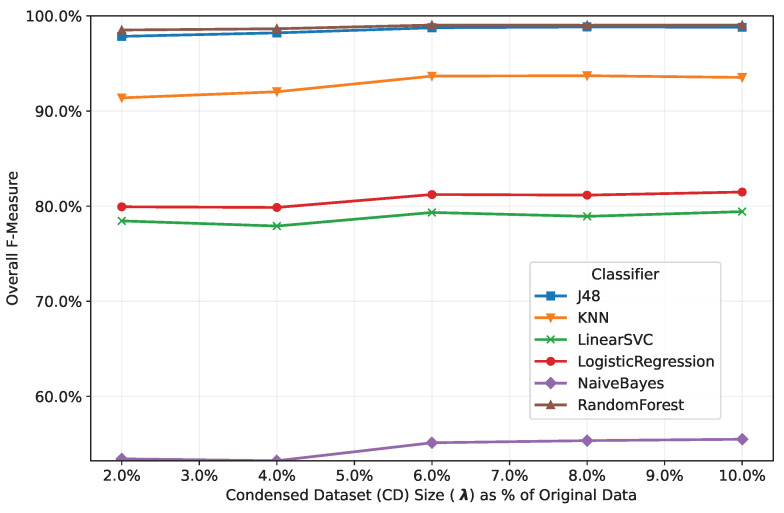
Classifiers’ classification accuracy vs. condensed dataset size across three datasets.

Figure 4 illustrates the impact of λ on the F-measure performance of the trained classifiers. To provide a generalized evaluation, we present the average F-measure values for each classifier across three IoT security datasets: N-BaIoT, Edge-IIoTset, and CICIoT2023. While increasing λ generally improves F-measure performance, our experiments reveal diminishing returns beyond λ=6 for these specific datasets. At this threshold, classifiers achieve comparable results to those with λ=8 or λ=10, suggesting a practical trade-off point under our experimental conditions. This finding highlights that optimal λ values are context-dependent and must be determined empirically. For IoT threat detection systems, our analysis identifies λ=6 as a cost-effective balance between dataset size and model efficiency in the studied scenarios. However, practitioners should validate this parameter experimentally for their own datasets and system constraints, as alternative environments may require different optimization strategies.
**Algorithm 3:** Representative observation selection.
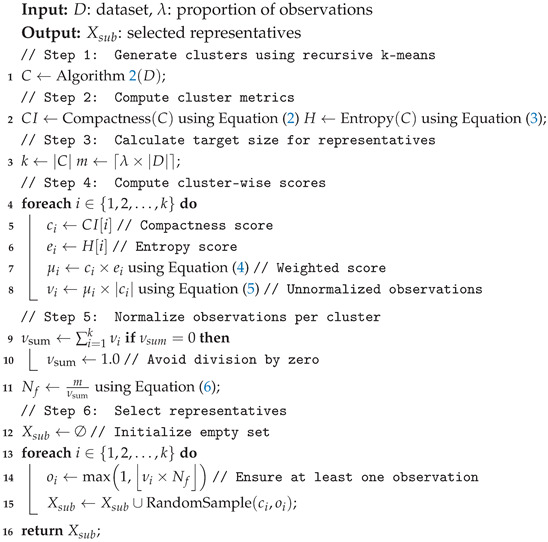


### 3.2. Dimensionality Reduction

This is the second fold of our proposed approach, which involves the selection of nearly optimal and stable features. In this part, we adapted the Monte Carlo Cross-Entropy optimization technique, coupled with a stability metric, to consistently identify and focus on the most stable features. Let $X_{\mathrm{sub}}$ be divided into *K* subsets, each denoted as $X_{\mathrm{sub},k}$ for k=1,2,…,K. For each subset, we apply the Monte Carlo Cross-Entropy optimization technique to select the best features. We generate $n_{k}$ independent binary samples. Each sample is a binary vector $S_{i}^{k}\in {\left\{0,1\right\}}^{d}$, where *d* is the total number of features. In this binary vector, $S_{i,j}^{k}=1$ indicates that the *j*-th feature is selected in the *i*-th sample for the *k*-th subset, and $S_{i,j}^{k}=0$ indicates that it is not selected, defined as $S_{i}^{k}=\left(S_{i,1}^{k},S_{i,2}^{k},\ldots ,S_{i,d}^{k}\right),\;i=1,2,\ldots ,n_{k},\;k=1,2,\ldots ,K$, where $n_{k}$ is the number of samples for the *k*-th subset. Each $S_{i}^{k}$ follows a Bernoulli distribution, and initially, each feature is assumed equally likely as $S_{i,j}^{k}∼\mathrm{Bernoulli}\left(p=0.5\right)$. Let β be the budget constraint, ensuring that no combination of features is generated with the number of selected features exceeding the threshold β. In the first iteration, the log-likelihood is not calculated because all samples are assumed equally likely. Starting from the second iteration, each generated sample $S_{i}^{k}$ is evaluated using the log-likelihood function to choose the best sample that represents the actual probabilities of each feature. The average log-likelihood of all samples is used as a threshold to select the candidate samples, denoted as $\psi_{k}$. The log-likelihood of each sample is calculated as follows:

(9) $$\begin{array}{c} \log L\left(S_{i}^{k}\right)=\sum_{j=1}^{N}S_{i,j}^{k}\log\left(p_{j,k}\right)\\ +\left(1-S_{i,j}^{k}\right)\log\left(1-p_{j,k}\right) \end{array}$$

The candidate samples are selected as follows:

(10) $$\psi_{k}=\left\{S_{i}^{k}|\log L\left(S_{i}^{k}\right)\geq \frac{1}{n_{k}}\sum_{i=1}^{n_{k}}\log L\left(S_{i}^{k}\right)\right\}$$

Each sample, which is an element of the candidate samples $\psi_{k}$, is evaluated using a fitness function $f(S_{i}^{k})$, and the samples are selected based on their contribution to accuracy. Therefore, we select the elite examples from the candidate samples $\psi_{k}$ when their accuracy is greater than or equal to the threshold ζ, defined as follows:

(11) $${\mathrm{Elite}}_{k}=\left\{S_{i}^{k}\in \psi_{k}|f\left(S_{i}^{k}\right)\geq \zeta \right\}$$

The probability of each feature is updated based on the normalized fitness scores of the elite samples, incorporating a learning rate η to ensure stability and prevent overfitting to a small number of elite samples in any iteration. This updating process can be defined as follows:

(12) $$\begin{array}{c} p_{\mathrm{new},k}=\left(1-\eta \right)\cdot p_{\mathrm{old},k}\;\\ +\eta \cdot \frac{\sum_{S_{i}^{k}\in {\mathrm{Elite}}_{k}}S_{i,j}^{k}\cdot f\left(S_{i}^{k}\right)}{\sum_{S_{i}^{k}\in {\mathrm{Elite}}_{k}}f\left(S_{i}^{k}\right)} \end{array}$$

The learning process continues by generating samples and evaluating them to identify nearly optimal features with high probabilities. This process stops when either of two conditions is met: the process reaches the maximum number of iterations, or the convergence between the new feature probabilities $p_{j,k}^{\mathrm{new}}$ and the previous ones $p_{j,k}^{\mathrm{old}}$ is sufficiently small, as indicated by a predefined threshold, ϵ. To monitor this convergence, we use the Kullback–Leibler (KL) divergence, which measures the difference between the updated and previous probability distributions. The KL divergence is computed as follows:

(13) $$\begin{array}{c} D_{\mathrm{KL}}\left(p_{j,k}^{\mathrm{old}}\;\|\;p_{j,k}^{\mathrm{new}}\right)=\sum_{j=1}^{N}\left(p_{j,k}^{\mathrm{old}}\log\left(\frac{p_{j,k}^{\mathrm{old}}}{p_{j,k}^{\mathrm{new}}}\right)\right.+\left.\left(1-p_{j,k}^{\mathrm{old}}\right)\log\left(\frac{1-p_{j,k}^{\mathrm{old}}}{1-p_{j,k}^{\mathrm{new}}}\right)\right) \end{array}$$

This divergence helps track whether the new probabilities are converging to a stable solution. When the divergence becomes smaller than ϵ, or when the maximum number of iterations has been reached, it indicates that the feature selection process has likely reached its optimal configuration. After the learning process has converged or reached the maximum iteration, the updated probabilities $p_{j,k}^{\mathrm{new}}$ represent the likelihoods of the features for subset *k*. These probabilities are then converted to binary values based on the threshold τ. A feature *j* is included in the feature set if $p_{j,k}^{\mathrm{new}}\geq \tau$ (set to 1) and excluded if $p_{j,k}^{\mathrm{new}}<\tau$ (set to 0). This results in the binary feature vector for subset *k*, denoted by $\Omega_{k}=\left(\Omega_{1,k},\Omega_{2,k},\ldots ,\Omega_{N,k}\right)$, where each $\Omega_{j,k}$ is presented as follows:

$$\Omega_{j,k}=\left\{\begin{array}{cc} 1 & \mathrm{if}\;p_{j,k}^{\mathrm{new}}\geq \tau \\ 0 & \mathrm{if}\;p_{j,k}^{\mathrm{new}}<\tau \end{array}\right.$$

The vector $\Omega_{k}$ represents the selected features for the *k*-th subset. The combined set of selected features across all subsets is denoted as $\Omega =(\Omega_{1},\Omega_{2},\ldots ,\Omega_{K})$, where each $\Omega_{k}$ is a binary feature vector for subset *k* learned via the Monte Carlo Cross-Entropy procedure. This collection aggregates the selected features across all subsets. To quantify stability, we compute a stability score for each feature *j*, defined as the proportion of times it is selected across the *K* subsets. This is calculated as follows:

$$\mathrm{Stability}\;\mathrm{Score}\left(j\right)=\frac{1}{K}\sum_{k=1}^{K}I\left(\Omega_{k}\left[j\right]=1\right),$$

where I(·) is an indicator function that equals 1 if feature *j* is selected in subset *k* (i.e., $\Omega_{k}\left[j\right]=1$) and 0 otherwise. The final stable features are then selected based on a stability threshold σ. This results in the final binary vector $\Omega_{\mathrm{stable}}$, where

(14) $$\Omega_{\mathrm{stable},j}=\left\{\begin{array}{cc} 1 & \mathrm{if}\;\mathrm{Stability}\;\mathrm{Score}(j)\geq \sigma \\ 0 & \mathrm{if}\;\mathrm{Stability}\;\mathrm{Score}(j)<\sigma \end{array}\right.\;\mathrm{for}\;j=1,2,\ldots ,N.$$

Thus, the vector $\Omega_{\mathrm{stable}}=\left(\Omega_{\mathrm{stable},1},\Omega_{\mathrm{stable},2},\ldots ,\Omega_{\mathrm{stable},N}\right)$ represents the final set of selected features after applying the stability threshold σ. The complete steps of the dimensionality reduction process are described in Algorithm 4.
**Algorithm 4:** Dimensionality reduction.
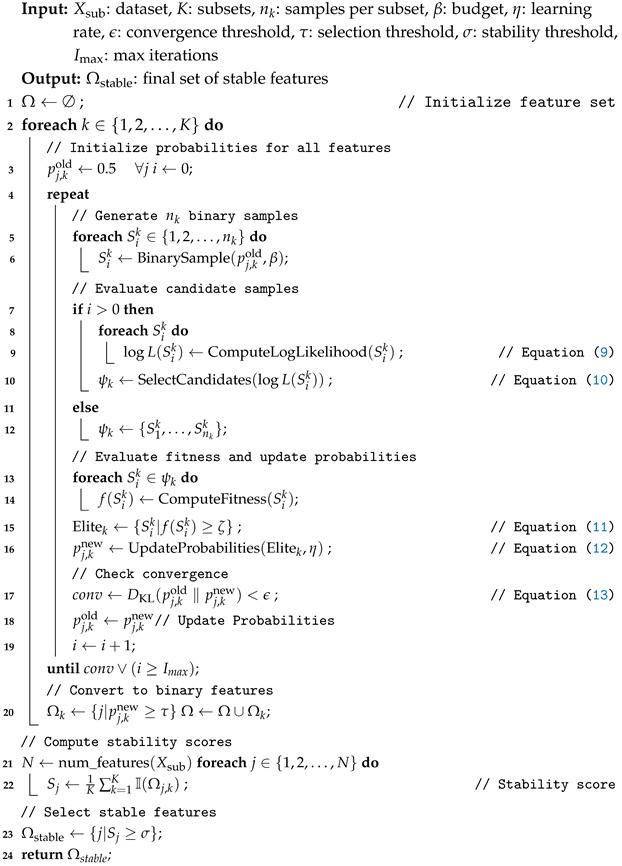


### 3.3. Complexity Analysis

We analyze the computational complexity of the proposed approach. The first stage employs recursive clustering. Unlike traditional *k*-means with complexity O(n·k·d·i), which is prohibitive for large *k* (e.g., 1000 clusters), where *n* is the number of data observations, *k* is the number of clusters, *d* is the number of dimensions, and *i* is the number of iterations, our approach achieves O(n·d·logn) complexity. This is achieved by forming a hierarchy through iterative splitting, starting with k=10 clusters (yielding an initial complexity of O(n·10·d·i)) and recursively splitting oversized clusters using a size threshold *T*. This creates a hierarchy of depth O(logn) when k=10 and *T* are fixed. Each split operates locally on cluster subsets, reducing per-iteration problem sizes (e.g., refining 1000 observations into 10 subclusters of 100 observations). The logarithmic depth and localized computations replace the linear *k*-dependence in *k*-means with a logarithmic dependence on *n*. While entropy and compactness calculations typically exhibit $O(n^{2})$ complexity, our approach bounds these costs by restricting computations to clusters of size m≤T. Finally, the feature selection process applies Monte Carlo Cross-Entropy on subsets of size $\frac{\gamma \cdot n}{k}$, enabling rapid convergence since fitness evaluations (e.g., classifier accuracy) occur on drastically reduced data. Here, *n* is the total dataset size, γ represents the percentage of *n*, and *k* is the number of subsets into which the representative dataset is divided.

**Remark 1.**  *A key advantage of the proposed approach is its inherent support for parallelism. Recursive clustering allows independent splitting of oversized clusters at each hierarchy level. Similarly, entropy and compactness calculations are confined to individual clusters (m≤T), enabling concurrent evaluations across all clusters. Feature selection further leverages parallelism by operating on disjoint subsets $\frac{\gamma \cdot n}{k}$, where Monte Carlo Cross-Entropy and fitness evaluations (e.g., classifier training) can be executed independently for each partition. This design eliminates synchronization barriers, enabling distributed processing of clusters and subsets, thereby scaling efficiently to large datasets.*

## 4. Evaluation

### 4.1. Training and Testing Data

In this study, we used three widely recognized datasets to evaluate security solutions tailored for IoT. These datasets capture two types of system behavior: normal and abnormal, with abnormal behavior representing various forms of cyber attacks. The first dataset, N-BaIoT [18], was generated from nine commercial IoT devices infected with the Mirai and BASHLITE botnets and is extensively used to study IoT vulnerabilities and evaluate mitigation strategies. The second dataset, Edge-IIoTset [19], is highly regarded within the cybersecurity community as a robust benchmark for evaluating IoT-based security solutions. It includes data from 14 distinct types of attacks specific to IoT and Industrial IoT protocols, categorized into five primary attack types. The third dataset, CICIoT2023 [20], is one of the most recent real-world datasets, generated from 105 IoT devices in a laboratory environment simulating real-world conditions. It is widely recognized within IoT security communities for its utility in developing and testing IoT security solutions.

### 4.2. The Selected Classifiers

We assessed the condensed dataset using six well-known classifiers that encompass a wide range of learning characteristics and perspectives, including probabilistic, tree-based, rule-based, and distance-based algorithms. A brief description of each is as follows:

1. LinearSVC is a discriminative model that employs an optimization algorithm known as “hinge loss” combined with L2 regularization [37]. The hinge loss penalizes both misclassified observations and those within the margin, while L2 regularization prevents overfitting by adding a penalty for large coefficients. LinearSVC utilizes the “liblinear” solver to find the optimal hyperplane that maximizes the margin between classes in high-dimensional data [38].
2. Naïve Bayes is a simple Bayesian classifier based on Bayes’ theorem, which assumes that features are independent of each other. It is widely used in many real-world applications, such as spam filtering, text classification, and intrusion detection systems. This classifier selects the class label with the highest probability, making it well suited for large databases and real-time applications due to its low computational cost [39].
3. J48 is a classification algorithm that employs a tree-based approach, building upon the C4.5 decision tree algorithm [40]. It constructs a tree-like structure leveraging the training data features, based on the concept of information gain. The algorithm recursively divides the dataset into subsets until each subset either belongs to a single class or can no longer be split further. Additionally, this classifier can handle both categorical and numerical data, and it is known for its robustness in dealing with noisy observations.
4. RandomForest is an ensemble learning classifier that constructs multiple decision trees using the training dataset. It achieves this by randomly selecting subsets of the training data through a process known as bootstrapping. Additionally, during the construction of each tree, random subsets of features are chosen at each split—a process known as feature bagging—which introduces another layer of randomness [41]. This random selection of features helps reduce correlation between the trees and mitigates overfitting by averaging the predictions of many diverse decision trees. As a result, random forests are more robust and less sensitive to noise in the training data, providing higher accuracy and better generalization compared to individual decision trees.
5. The K-Nearest Neighbor (KNN) is a simple, non-parametric algorithm widely used for both classification and regression tasks. This algorithm classifies a query observation based on its “k” nearest neighbors, using the majority class for classification or the average for regression. While KNN can achieve high accuracy, it is computationally expensive as the size of the training dataset increases. To address this, various optimization techniques, such as KD-trees, Ball-trees, etc. [42], have been developed to speed up the search process and reduce the need to examine the entire dataset. The performance of KNN is influenced by the choice of “k” and the distance metric, with traditional KNN often relying on the Euclidean distance to determine neighborhood boundaries.
6. Logistic Regression is a statistical method widely used for both binary and multiclass classification problems. In its simplest form, it models the relationship between input features and the probability of a specific outcome using the logistic function, also known as the sigmoid function, which maps any real-valued number into the range [0, 1]. For multiclass classification, logistic regression can be extended using techniques such as “one-vs-rest” or “softmax regression”. During training, logistic regression learns decision boundaries to separate classes by calculating the weights and bias, which are optimized using algorithms like Gradient Descent. In the prediction phase, the model uses the calculated probabilities to predict the class of new observations.

### 4.3. Performance Evaluation Metrics

The metrics utilized for evaluating the performance of a classification approach include Precision, Recall, and the F-measure (also known as the F-score). These metrics provide a quantitative assessment of the model’s ability to correctly identify class labels, regardless of the size of the training and testing datasets. Recall is calculated as the ratio of correctly identified class labels (True Positives, TP) to the sum of True Positives and class labels that were not identified or misclassified (False Negatives, FN). This is defined as follows:

$$\mathrm{Recall}=\frac{\mathrm{TP}}{\mathrm{TP}+\mathrm{FN}}$$

Precision, on the other hand, measures the accuracy of positive predictions by dividing the number of correctly identified positive class labels (TP) by the total number of positive predictions made by the approach, including False Positives (FP). The formula for Precision is as follows:

$$\mathrm{Precision}=\frac{\mathrm{TP}}{\mathrm{TP}+\mathrm{FP}}$$

The F-measure, or F-score, merges Precision and Recall into one metric by computing their harmonic mean. This measure is highly useful in situations with class imbalance or when there is a need to weigh Precision and Recall equally. The F-measure is given by the following:

$$F\mathrm{-measure}=2\times \frac{\mathrm{Precision}\times \mathrm{Recall}}{\mathrm{Precision}+\mathrm{Recall}}$$

### 4.4. Experimental Settings

In this section, we elaborate on the experimental settings established for the experiment. As mentioned earlier, three datasets are used to evaluate the proposed approach. The experiment is not conducted on the entire datasets; instead, sampling is performed to select subsets from each dataset. Table 2 provides a summary of the sampled datasets derived from the original ones. As discussed earlier, the proposed approach consists of two parts: the representative selection and feature selection processes, each requiring a specific set of settings. In the representative selection, we set the initial number of clusters in each clustering process to 10, and the threshold for the maximum cluster size is set to 2000. The weighting factor, α, is set to 0.5 to balance the trade-off between compactness and entropy. Additionally, λ is set to 6%, meaning that representative observations comprise 6% of the entire training dataset. All methods are implemented in Python 3.12.9 using Scikit-learn and executed on a single core of an Intel Core i9-12900HK (2.50 GHz) CPU with 32 GB RAM on Windows 11 Pro. The implementation utilizes the Scikit-learn library, including modules for classification, feature selection, data preprocessing, and evaluation.

On the other hand, the feature selection component has a set of parameters; however, based on the experimental results, these parameters are not highly sensitive. The settings used in this evaluation are as follows: the feature selection parameters include Epsilon (ϵ=0.01) as the convergence threshold, Tau (τ=0.5) and Eta (η=0.5) as thresholds controlling the selection process, a budget of 20 features, MaxIterations = 100, N = 200 samples per iteration, and ζ = 0.97 as the probability threshold. For more details on each threshold, see Algorithm 4. The training dataset is split into K = 5 subsets to work independently on each one to evaluate the stability of features, and σ is set to 0.5 to decide the final feature evaluation based on stability. The fitness metric used in the feature selection process is evaluated using J48, which is more efficient in both training and testing, to assess the performance of the selected features. Notably, these settings are chosen after extensive experimentation, and while the results are not shown here due to space constraints, they represent the optimal values for this experiment.

## 5. Experimental Results and Comparisons

In this section, we present a comprehensive evaluation to assess how effectively the condensed dataset retains the essential characteristics of the original dataset using three distinct datasets (described in Section 4.1), each partitioned into training and testing subsets as specified in Table 2. Our proposed method generates the condensed data, which are benchmarked against two baselines: (i) the full data, representing the original unreduced dataset, and (ii) the feature-selected data, produced by the feature selection method proposed in [30]. The latter employs four ridge regression-based techniques—importance-coefficient, correlation-coefficient, forward-sequential, and backward-sequential selection—to derive a reduced feature subset. Among these techniques, we focus on the correlation-coefficient method for comparison, as it achieved the optimal balance between accuracy and computational efficiency in preliminary experiments. For consistency, the feature-selected data retain the top 15 features, a configuration that maximized detection performance while minimizing computational overhead. For simplicity, we refer to the full, condensed, and feature-selected datasets as Full, CD, and FSD, respectively.

In this evaluation, six well-established classifiers spanning diverse learning paradigms (see Section 4.2) were trained as classification-based IDS models. The comparison focuses on three critical dimensions. First, we measure classification performance to determine how accurately each dataset supports intrusion detection. Second, we analyze the computational efficiency of training and testing times, thereby assessing the impact of dataset size on learning and prediction speeds. Third, we evaluate the memory usage of models trained on each dataset. This analysis highlights the trade-off between detection accuracy, computational cost, and memory requirements, providing insights into the practicality of Full, CD and FSD for IDS deployment.

### 5.1. ClassificationPerformance Analysis

Here, we evaluate the classification accuracy of CD (generated by our proposed approach) by comparing it to both Full and FSD. We begin with the classification results for the N-BaIoT dataset. Table 3, Table 4 and Table 5 summarize the results for Full, CD, and FSD, corresponding to precision, recall, and F-measure, respectively.

As illustrated by Table 3, the precision values across the Full, CD, and FSD in most classifiers maintain strong performance in class labels such as ack, combo, and normal. For instance, J48 achieves perfect precision (1.00) on ack, combo, and several other class labels under both Full and FSD conditions. RandomForest similarly exhibits consistently high precision, often reaching 1.00 for the same class labels across all three dataset versions. KNN, on the other hand, displays more variability—particularly on ack, where its precision drops from 0.96 under Full to 0.75 in CD, but rises again to 1.00 in FSD. LinearSVC and NaiveBayes show slightly lower precision for certain traffic types (e.g., tcp, junk), yet their performances remain relatively close when comparing Full, CD, and FSD. These findings suggest that, although precision can dip for specific class labels and classifiers when the dataset is condensed or feature-selected, the overall classification capability remains robust.

Similarly to the precision results, Table 4 presents the recall values on the N-BaIoT dataset for the Full, CD and FSD versions, indicating how effectively each model identifies all positive observations within a class. Here, RandomForest and J48 again dominate, often reaching or hovering near 1.00 for normal, scan, and syn across Full, CD, and FSD. KNN also tends to achieve high recall for these classes, though it shows notable reductions in the CD version for ack (e.g., dropping from 0.93 in Full to 0.67 in CD). NaiveBayes’ recall varies more widely depending on the class and data reduction method: while it maintains high recall for ack in Full (1.00), it dips to 0.44 in CD but rebounds to 1.00 in FSD. Meanwhile, classes such as tcp and udp remain difficult for multiple classifiers, likely reflecting the inherent complexity of these traffic types rather than the data size. Overall, although some minor declines appear in the condensed or feature-selected variants, most recall values remain close to those achieved in the Full dataset.

Similar to the precision and recall results, Table 5 focuses on F-measure, which combines precision and recall to provide a balanced view of each classifier’s overall performance. Across most classes, RandomForest and J48 achieve high F-measure values—even reaching 1.00 in some cases—with minimal degradation when transitioning from Full to CD or FSD. NaiveBayes generally demonstrates moderate F-measures; for example, its performance on the ack class is 0.48 in Full, 0.42 in CD, and 0.47 in FSD, indicating it remains fairly competitive in each version. By contrast, classes such as tcp tend to yield lower F-measures for multiple models, underscoring persistent classification challenges irrespective of data size or feature selection. Nonetheless, the relatively narrow gaps between Full, CD, and FSD show that both data condensation and feature selection can significantly reduce dataset volume while preserving sufficient information for accurate classification.

Next, we analyze the results of CICIoT2023 dataset, applying the same evaluation framework. As illustrated by Table 6, the precision values across Full, CD, and FSD versions for the CICIoT2023 dataset vary noticeably among classifiers and classes. For instance, J48 maintains perfect precision (1.00) on DDoS, DoS, and Mirai under both Full and CD conditions, yet these values decline in FSD for DDoS (0.89) and DoS (0.67). RandomForest exhibits similarly high precision in DDoS, DoS, and Mirai (frequently around 1.00) across all three dataset versions, underscoring its robustness. By contrast, Web-based attacks often yield lower precision, with KNN and LinearSVC posting especially modest numbers in both CD and FSD. Another notable trend emerges in the Brute Force class, where J48’s precision drops from 0.62 (Full) to 0.13 (FSD), while NaiveBayes struggles to classify Brute Force accurately under all dataset versions. Overall, most classifiers remain relatively stable in precision for frequent or well-represented classes (DDoS, DoS, Mirai), but some classes (Brute Force, Web-based) see more pronounced performance dips, particularly when the dataset is condensed or feature-reduced.

Table 7 shows the recall values, reflecting each classifier’s ability to identify all positive observations of a given class. Here, again, RandomForest and J48 stand out with high recall for DDoS and DoS under Full and CD, though DoS experiences a substantial drop in *FSD* (e.g., 0.53 for J48). Mirai remains consistently easier to detect across all classifiers and dataset versions, often nearing 1.00 in recall. Meanwhile, Spoofing and Brute Force prove more challenging, as many classifiers display lower recall or even fail entirely under CD or FSD (e.g., KNN’s recall for Brute Force hovers near 0.10 or less in those reduced datasets). Such patterns likely indicate that these classes are either more complex or less represented, making them harder to classify once data volume or dimensionality is reduced.

Finally, Table 8 combines precision and recall to provide a balanced perspective on overall performance. RandomForest and J48 generally maintain the highest F-measure scores for several frequent classes (e.g., DDoS, Mirai, DoS), often retaining strong performance from Full to CD, though certain classes (like DoS in FSD) show noticeable declines. In contrast, classifiers such as LinearSVC and NaiveBayes exhibit more dramatic shifts in F-measure for classes like Brute Force and Spoofing, reflecting lower consistency in identifying these attacks once features or data points are reduced. Nonetheless, Mirai remains consistently well classified, indicating that even a condensed or feature-selected dataset can retain sufficient information for some attack types. Overall, these findings suggest that while condensing and feature selection methods can lead to certain drops in classification metrics—especially for trickier, less frequent classes—strong models (notably RandomForest and J48) can largely preserve high accuracy across the CICIoT2023 dataset.

Finally, we present the results for the Edge-IIoTset dataset. As shown in Table 9, the precision values across Full, CD, and FSD for the Edge-IIoTset dataset vary considerably by both class label and classifier. Certain attacks, such as DDoS_ICMP and DDoS_TCP, often achieve near-perfect precision under J48 and RandomForest across all dataset versions—indicating that these attack types are relatively easier to distinguish. In contrast, XSS and SQL_injection pose more challenges: J48’s precision for XSS declines from 0.55 (Full) to 0.18 (FSD), and LinearSVC and LogisticRegression record zero precision for SQL_injection under certain reduced conditions. Despite these dips for specific classes, RandomForest generally displays robust precision (e.g., near 1.00 on multiple DDoS variants), suggesting that it copes better with CD and FSD.

Turning to recall in Table 10, we see a similar pattern of strong performance on classes like DDoS_ICMP and DDoS_TCP, where many classifiers (particularly J48, RandomForest) maintain recall values close to 1.00. Normal traffic likewise remains consistently identifiable. However, attacks such as XSS and OS_Fingerprinting become notably more difficult once data are condensed or feature-selected; LinearSVC, for instance, records 0.00 recall for XSS and OS_Fingerprinting in some reduced scenarios. Another notable example is Ransomware, for which multiple classifiers’ recall drops significantly in CD and FSD conditions, indicating that these attacks may require more features or data volume for reliable detection.

Similarly, we now analyze the F-measure results, which are presented in Table 11. J48 and RandomForest again excel on DDoS_ICMP and DDoS_TCP, frequently preserving F-measures near 1.00. Even for challenging classes, J48 and RandomForest generally retain moderate to high F-measures between Full and CD, though XSS and Ransomware remain problematic in most classifiers once the dataset is reduced. In addition, LinearSVC and LogisticRegression exhibit significant drops for certain classes (e.g., Port_Scanning, Password in FSD), reflecting their susceptibility to reduced feature sets. In general, while data condensation and feature selection can lead to declines in classification metrics for more complex or less frequent attacks, the consistently high scores for many DDoS variants imply that effective detection can still be achieved using a smaller feature space—provided the chosen model (e.g., RandomForest or J48) is robust enough to handle these constraints.

Table 12 presents a consolidated summary of classification accuracy using a 90% acceptability threshold. This summary is derived from the detailed classification accuracy results reported in Table 3, Table 4, Table 5, Table 6, Table 7, Table 8, Table 9, Table 10 and Table 11, providing an overall assessment of how well each approach (CD or FSD) maintains performance compared to the Full model. An approach is considered acceptable for a class label if its performance (precision, recall, F-measure) meets or exceeds 90% of the Full model’s score across all three metrics for that classifier and label. For example, the N-BaIoT dataset has nine class labels, and each classifier is evaluated on all labels. A value like 8/9 for CD and J48 indicates that eight class labels achieved acceptable performance when trained on the reduced CD dataset. Therefore, Table 12 provides a high-level comparison, quantifying how consistently each approach preserves near-original performance per classifier and dataset at the class level.

Overall, Table 12 shows that the proposed approach, CD, preserves much of the original performance—especially for J48 and RandomForest—while also providing an opportunity to balance classification accuracy against runtime and resource constraints. However, increasing the observations and features in the CD improves accuracy at the expense of heightened memory usage and computational overhead. Thus, selecting the appropriate CD becomes a trade-off that should be tailored to each specific environment and operational requirement.

### 5.2. Runtime Performance

This section explores the impact of (Full/CD/FSD) on the runtime performance of the classifiers in terms of both training and testing time, providing insights into the computational efficiency across different datasets. The results in Table 13 indicate that the dataset generated using our proposed approach, referred to as *CD*, significantly reduces training time across all six classifiers compared to both the Full and FSD. This efficiency is especially clear in the N-BaIoT dataset, where *CD* consistently outperforms the other datasets, achieving notably faster training times. A similar trend is observed with the CICIoT2023 and Edge-IIoTset datasets, where CD remains the most efficient and considerably lowers the computational cost relative to the Full and FSD. Such efficiency is particularly promising for the development of lightweight intrusion detection systems (IDSs) that are quicker and easier to train. Moreover, these improvements in training time also have a positive impact on testing performance, which is presented in Table 14.

Following the discussion on training time, we now analyze the impact on testing performance, as shown in Table 14. The results demonstrate that CD continues to outperform both Full and FSD across nearly all classifiers and datasets. In particular, CD consistently achieves the lowest mean testing time, highlighting its efficiency in reducing computational overhead. However, in the CICIoT2023 dataset, two exceptions are observed: for LinearSVC and Naïve Bayes, FSD exhibits slightly better testing times in a few cases. Despite this, the difference between CD and FSD remains minimal, indicating that CD remains competitive in these observations.

### 5.3. RAM Usage

In this final evaluation, we explore the impact of (Full/CD/FSD) on the RAM usage of classifiers across different datasets. As can be seen in Table 15, the results highlight the impact of Full, CD, and FSD on memory consumption across the N-BaIoT, CICIoT2023, and Edge-IIoTset IoT datasets. In the N-BaIoT dataset, CD consistently achieved the lowest RAM usage for classifiers such as J48, KNN, and Logistic Regression, while remaining highly competitive with FSD for RandomForest. Similarly, in the CICIoT2023 dataset, CD generally maintained the lowest memory usage, with FSD slightly outperforming it only in LinearSVC and Naïve Bayes. In the Edge-IIoTset dataset, although CD remained competitive, classifiers like Naïve Bayes and RandomForest consumed more RAM than with FSD, possibly due to the broader range of features retained in the condensed version. Unlike FSD, which strictly reduces features, CD condenses observations while keeping more features, potentially influencing memory usage in feature-sensitive classifiers. Overall, these findings suggest that CD effectively reduces RAM consumption—often outperforming FSD—yet the performance can vary depending on the classifier and dataset, particularly when feature dimensionality plays a significant role. Consequently, while CD offers substantial memory savings and is advantageous for large-scale IoT datasets, attention should be given to classifiers like Naïve Bayes and RandomForest, which rely heavily on feature distributions and may benefit more from strictly reducing features.

## 6. Conclusions

This paper presents a novel lightweight intrusion detection system (IDS) designed for resource-constrained IoT environments, addressing the growing need for efficient security solutions in response to escalating cyber threats. Our approach combines recursive clustering for data condensation with the Monte Carlo Cross-Entropy method for feature selection. This ensures the selection of representative observations to reduce computational overhead while maintaining high detection accuracy. Evaluations on realistic IoT datasets generated from real devices demonstrate that our approach achieves detection accuracy comparable to models trained on full and feature-reduced datasets, while significantly reducing training/testing times and memory usage, making it ideal for resource-limited IoT devices. In future work, we aim to further reduce the condensed dataset by selecting representatives based not only on local cluster characteristics but also on their global contribution to the dataset or cluster neighborhoods, ensuring minimal size with maximum representativeness. The proposed lightweight IDS offers numerous advantages, including resource-efficient processing suitable for resource-constrained IoT devices. Simultaneously, it maintains classification accuracy in IoT environments while requiring minimal resources, enabling scalability and adaptability to diverse settings. Furthermore, the model can be deployed across various IoT environments, such as edge devices, cloud-based security platforms, smart home security systems, and healthcare device protection. However, to validate the efficiency of our approach in real-world scenarios, future work will involve testing on IoT testbeds and edge computing frameworks to assess practical feasibility, limitations, and challenges.

## Figures and Tables

**Table 1:** List of acronyms.

Acronym	Full Form
IoT	Internet of Things
IDS	Intrusion Detection System
ML	Machine Learning
DoS	Denial of Service
DDoS	Distributed Denial of Service
IIoT	Industrial Internet of Things
CE	Cross-Entropy
IC	Intra-Cluster Compactness
SES	Spatial Entropy Score
KL	Kullback–Leibler (Divergence)
FSD	Feature-Selected Data
CD	Condensed Dataset
RAM	Random Access Memory
KNN	K-Nearest Neighbor
SVM	Support Vector Machine
TCP/IP	Transmission Control Protocol/Internet Protocol
N-BaIoT	Network-Based IoT Botnet Dataset
Edge-IIoTset	Edge-Industrial IoT Dataset
CICIoT2023	Collaborative and Intelligent Cybersecurity for IoT 2023 Dataset
FL	Federated Learning

**Table 2:** Overview of training and testing datasets.

Dataset	Total	Normal	Abnormal	Attack Types	Features
Edge-IIoTset-Training	525,273	335,606	189,667	13	70
Edge-IIoTset-Testing	225,117	143,831	81,286	13	70
N-BaIoT-Training	146,618	61,566	85,052	8	115
N-BaIoT-Testing	62,837	26,386	36,451	8	115
CICIoT2023-Training	352,282	7687	344,595	7	46
CICIoT2023-Testing	150,978	3294	147,684	7	46

**Table 3:** Classification performance based on precision for the N-BaIoT dataset.

Class Label	J48			KNN			LinearSVC			LogisticRegression			NaiveBayes			RandomForest		
	Full	CD	FSD	Full	CD	FSD	Full	CD	FSD	Full	CD	FSD	Full	CD	FSD	Full	CD	FSD
ack	1.00	0.99	1.00	0.96	0.75	1.00	0.95	0.41	0.36	0.96	0.43	0.48	0.32	0.40	0.31	1.00	1.00	1.00
combo	1.00	0.96	1.00	0.98	0.91	0.93	0.81	0.66	0.65	0.80	0.67	0.74	0.71	0.76	0.68	1.00	0.98	1.00
junk	1.00	0.94	1.00	0.95	0.93	0.86	0.61	0.42	0.52	0.60	0.53	0.49	0.50	0.58	0.03	1.00	0.97	1.00
normal	1.00	1.00	1.00	1.00	1.00	1.00	1.00	0.99	0.99	1.00	0.99	0.98	1.00	1.00	1.00	1.00	1.00	1.00
scan	1.00	1.00	1.00	0.99	0.99	1.00	0.99	0.95	0.82	0.99	0.98	0.77	0.27	0.40	0.18	1.00	1.00	1.00
syn	1.00	1.00	1.00	1.00	0.96	1.00	1.00	0.77	0.72	0.99	0.89	0.84	0.53	0.78	0.47	1.00	1.00	1.00
tcp	0.38	0.20	0.67	0.50	0.00	1.00	0.25	0.00	0.00	0.20	0.00	0.00	0.00	0.00	0.01	0.40	1.00	0.67
udp	1.00	0.99	1.00	0.97	0.85	1.00	0.88	0.64	0.64	0.93	0.67	0.67	0.43	0.67	0.01	1.00	1.00	1.00
udpplain	1.00	0.98	1.00	0.99	0.91	1.00	0.97	0.86	0.97	0.97	0.84	0.96	0.95	0.61	0.87	1.00	1.00	1.00

**Table 4:** Classification performance based on recall for the N-BaIoT dataset.

Class Label	J48			KNN			LinearSVC			LogisticRegression			NaiveBayes			RandomForest		
	Full	CD	FSD	Full	CD	FSD	Full	CD	FSD	Full	CD	FSD	Full	CD	FSD	Full	CD	FSD
ack	1.00	0.99	1.00	0.93	0.67	1.00	0.75	0.18	0.20	0.86	0.22	0.27	1.00	0.44	1.00	1.00	1.00	1.00
combo	1.00	0.97	1.00	0.98	0.94	0.93	0.79	0.75	0.74	0.79	0.75	0.71	0.90	0.79	0.81	1.00	0.98	1.00
junk	1.00	0.93	0.99	0.95	0.89	0.86	0.64	0.28	0.26	0.61	0.41	0.32	0.04	0.17	0.02	1.00	0.97	1.00
normal	1.00	1.00	1.00	1.00	1.00	1.00	1.00	1.00	1.00	1.00	1.00	1.00	0.63	0.66	0.51	1.00	1.00	1.00
scan	1.00	1.00	1.00	1.00	1.00	1.00	1.00	0.83	0.69	1.00	0.96	0.89	0.37	0.97	0.33	1.00	1.00	1.00
syn	1.00	1.00	1.00	1.00	0.95	1.00	1.00	0.86	0.88	0.99	0.87	0.80	1.00	1.00	1.00	1.00	1.00	1.00
tcp	0.27	0.18	0.73	0.09	0.00	0.09	0.18	0.00	0.00	0.09	0.00	0.00	0.55	0.00	0.27	0.18	0.09	0.55
udp	1.00	0.99	1.00	0.98	0.89	1.00	0.97	0.90	0.90	0.98	0.84	0.85	0.00	0.66	0.00	1.00	1.00	1.00
udpplain	1.00	0.99	1.00	1.00	0.94	1.00	1.00	0.64	0.63	0.99	0.87	0.88	0.62	0.59	0.62	1.00	1.00	1.00

**Table 5:** Classification performance based on F-measure for the N-BaIoT dataset.

Class Label	J48			KNN			LinearSVC			LogisticRegression			NaiveBayes			RandomForest		
	Full	CD	FSD	Full	CD	FSD	Full	CD	FSD	Full	CD	FSD	Full	CD	FSD	Full	CD	FSD
ack	1.00	0.99	1.00	0.94	0.71	1.00	0.83	0.25	0.25	0.91	0.29	0.34	0.48	0.42	0.47	1.00	1.00	1.00
combo	1.00	0.97	1.00	0.98	0.93	0.93	0.80	0.70	0.69	0.80	0.71	0.72	0.79	0.77	0.74	1.00	0.98	1.00
junk	1.00	0.93	0.99	0.95	0.91	0.86	0.63	0.34	0.35	0.61	0.46	0.39	0.07	0.26	0.03	1.00	0.97	1.00
normal	1.00	1.00	1.00	1.00	1.00	1.00	1.00	0.99	1.00	1.00	0.99	0.99	0.78	0.80	0.67	1.00	1.00	1.00
scan	1.00	1.00	1.00	1.00	1.00	1.00	0.99	0.89	0.75	1.00	0.97	0.83	0.31	0.57	0.23	1.00	1.00	1.00
syn	1.00	1.00	1.00	1.00	0.95	1.00	1.00	0.82	0.79	0.99	0.88	0.82	0.70	0.88	0.64	1.00	1.00	1.00
tcp	0.32	0.19	0.70	0.15	0.00	0.17	0.21	0.00	0.00	0.12	0.00	0.00	0.00	0.00	0.01	0.25	0.17	0.60
udp	1.00	0.99	1.00	0.97	0.87	1.00	0.93	0.75	0.75	0.95	0.75	0.75	0.00	0.66	0.00	1.00	1.00	1.00
udpplain	1.00	0.99	1.00	0.99	0.93	1.00	0.98	0.73	0.77	0.98	0.86	0.92	0.75	0.60	0.72	1.00	1.00	1.00

**Table 6:** Classification performance based on precision for the CICIoT2023 dataset.

Class Label	J48			KNN			LinearSVC			LogisticRegression			NaiveBayes			RandomForest		
	Full	CD	FSD	Full	CD	FSD	Full	CD	FSD	Full	CD	FSD	Full	CD	FSD	Full	CD	FSD
BenignTraffic	0.84	0.81	0.57	0.56	0.50	0.54	0.56	0.45	0.52	0.56	0.54	0.55	0.22	0.39	0.32	0.85	0.82	0.63
Brute Force	0.62	0.55	0.13	0.33	0.11	0.17	1.00	0.00	0.00	1.00	0.00	0.00	0.37	0.02	0.00	0.94	0.79	0.46
DDoS	1.00	1.00	0.89	0.97	0.97	0.89	0.81	0.82	0.79	0.81	0.83	0.80	0.99	0.98	1.00	1.00	1.00	0.90
DoS	1.00	1.00	0.67	0.91	0.76	0.65	0.73	0.36	0.20	0.78	0.33	0.80	0.31	0.21	0.27	1.00	1.00	0.71
Mirai	1.00	1.00	0.99	1.00	0.99	1.00	0.97	0.90	0.96	0.98	0.95	0.98	1.00	0.10	1.00	1.00	1.00	1.00
Recon	0.86	0.83	0.63	0.66	0.63	0.61	0.54	0.53	0.45	0.54	0.45	0.44	0.87	0.91	0.46	0.84	0.82	0.64
Spoofing	0.82	0.80	0.58	0.71	0.58	0.69	0.79	0.59	0.66	0.62	0.62	0.70	0.80	0.58	0.68	0.86	0.81	0.75
Web-based	0.73	0.70	0.34	0.44	0.31	0.36	0.23	0.00	0.13	0.36	0.00	0.13	0.30	0.04	0.10	0.73	0.72	0.47

**Table 7:** Classification performance based on recall for the CICIoT2023 dataset.

Class Label	J48			KNN			LinearSVC			LogisticRegression			NaiveBayes			RandomForest		
	Full	CD	FSD	Full	CD	FSD	Full	CD	FSD	Full	CD	FSD	Full	CD	FSD	Full	CD	FSD
BenignTraffic	0.84	0.81	0.57	0.70	0.60	0.67	0.64	0.55	0.46	0.63	0.49	0.47	0.97	0.59	0.51	0.90	0.88	0.72
Brute Force	0.67	0.56	0.13	0.19	0.10	0.10	0.15	0.00	0.00	0.15	0.00	0.00	0.18	0.90	0.00	0.48	0.44	0.08
DDoS	1.00	1.00	0.94	0.98	0.93	0.93	0.99	0.83	0.99	0.99	0.82	0.99	0.46	0.12	0.34	1.00	1.00	0.94
DoS	1.00	1.00	0.53	0.87	0.90	0.51	0.12	0.40	0.00	0.12	0.39	0.04	0.95	0.25	0.96	1.00	1.00	0.55
Mirai	1.00	1.00	0.99	0.99	0.96	0.99	0.99	0.94	0.99	0.98	0.90	0.98	0.99	0.99	0.99	1.00	1.00	0.99
Recon	0.86	0.84	0.62	0.71	0.68	0.69	0.60	0.37	0.61	0.61	0.45	0.65	0.21	0.20	0.02	0.88	0.87	0.75
Spoofing	0.82	0.78	0.59	0.58	0.54	0.52	0.34	0.23	0.25	0.50	0.25	0.31	0.06	0.27	0.16	0.80	0.78	0.61
Web-based	0.73	0.70	0.35	0.33	0.16	0.24	0.01	0.00	0.01	0.04	0.00	0.01	0.34	0.01	0.80	0.72	0.61	0.39

**Table 8:** Classification performance based on F-measure for the CICIoT2023 dataset.

Class Label	J48			KNN			LinearSVC			LogisticRegression			NaiveBayes			RandomForest		
	Full	CD	FSD	Full	CD	FSD	Full	CD	FSD	Full	CD	FSD	Full	CD	FSD	Full	CD	FSD
BenignTraffic	0.84	0.81	0.57	0.62	0.54	0.60	0.60	0.49	0.49	0.59	0.51	0.51	0.36	0.47	0.39	0.87	0.85	0.67
Brute Force	0.64	0.55	0.13	0.24	0.11	0.12	0.26	0.00	0.00	0.26	0.00	0.00	0.24	0.03	0.00	0.63	0.56	0.14
DDoS	1.00	1.00	0.91	0.97	0.95	0.91	0.89	0.82	0.88	0.89	0.82	0.88	0.63	0.22	0.50	1.00	1.00	0.92
DoS	1.00	1.00	0.59	0.89	0.83	0.57	0.20	0.38	0.00	0.20	0.36	0.08	0.47	0.23	0.42	1.00	1.00	0.62
Mirai	1.00	1.00	0.99	1.00	0.98	1.00	0.98	0.92	0.98	0.98	0.92	0.98	0.99	0.18	0.99	1.00	1.00	1.00
Recon	0.86	0.83	0.63	0.69	0.65	0.65	0.57	0.44	0.52	0.57	0.45	0.53	0.33	0.33	0.03	0.86	0.85	0.69
Spoofing	0.82	0.79	0.59	0.64	0.56	0.60	0.48	0.33	0.36	0.55	0.36	0.43	0.11	0.37	0.26	0.83	0.79	0.68
Web-based	0.73	0.70	0.34	0.37	0.21	0.29	0.02	0.00	0.01	0.08	0.00	0.01	0.32	0.02	0.18	0.73	0.66	0.43

**Table 9:** Classification performance based on precision for the Edge-IIoTset dataset.

Class Label	J48			KNN			LinearSVC			LogisticRegression			NaiveBayes			RandomForest		
	Full	CD	FSD	Full	CD	FSD	Full	CD	FSD	Full	CD	FSD	Full	CD	FSD	Full	CD	FSD
Backdoor	0.98	0.91	0.58	0.95	0.69	0.24	0.82	0.00	0.00	0.83	0.00	0.00	0.05	0.00	0.44	0.99	1.00	0.65
DDoS_HTTP	1.00	1.00	0.87	1.00	1.00	0.83	0.77	0.03	0.42	0.83	0.56	0.54	1.00	1.00	0.00	1.00	1.00	0.84
DDoS_ICMP	1.00	1.00	1.00	0.99	0.99	1.00	0.99	0.98	0.97	0.99	0.97	0.97	1.00	1.00	0.97	1.00	1.00	1.00
DDoS_TCP	1.00	1.00	1.00	1.00	0.98	1.00	1.00	0.88	0.80	1.00	0.90	0.84	0.96	0.95	0.56	1.00	1.00	1.00
DDoS_UDP	1.00	1.00	0.50	1.00	1.00	0.50	0.60	1.00	0.00	1.00	0.00	0.00	1.00	1.00	0.50	1.00	1.00	0.50
Normal	1.00	1.00	1.00	1.00	1.00	1.00	0.98	0.92	0.81	0.98	0.95	0.84	1.00	1.00	0.90	1.00	1.00	1.00
OS_Fingerprinting	0.99	0.96	0.84	0.83	0.71	0.59	1.00	0.00	0.00	0.80	0.00	0.00	0.63	0.67	0.00	0.99	0.92	0.84
Password	0.98	0.95	0.89	0.88	0.88	0.82	0.72	0.53	0.60	0.79	0.57	0.63	0.43	0.16	0.99	0.95	0.95	0.87
Port_Scanning	0.96	0.94	0.82	0.93	0.91	0.92	0.85	0.00	1.00	0.93	0.00	0.99	0.09	0.29	0.06	0.96	0.97	0.84
Ransomware	0.99	0.94	0.75	0.89	0.73	0.57	0.63	0.44	0.00	0.61	0.32	0.00	0.04	0.63	0.03	0.98	0.95	0.73
SQL_injection	0.99	1.00	0.46	0.80	0.80	0.39	0.50	0.00	0.00	0.16	0.00	0.00	0.80	0.61	0.25	1.00	1.00	0.57
Uploading	0.99	0.91	0.64	0.93	0.83	0.69	0.88	0.83	0.00	0.83	0.81	0.83	0.94	0.79	0.44	1.00	0.98	0.77
Vulnerability_scanner	0.97	0.89	0.91	0.93	0.71	0.93	0.94	0.00	0.92	0.95	0.00	0.93	0.94	0.38	0.80	1.00	0.94	0.98
XSS	0.55	0.21	0.18	0.67	0.00	0.89	0.51	0.00	0.00	1.00	0.00	0.00	0.71	0.01	0.00	0.88	0.21	0.65

**Table 10:** Classification performance based on recall for the Edge-IIoTset dataset.

Class Label	J48			KNN			LinearSVC			LogisticRegression			NaiveBayes			RandomForest		
	Full	CD	FSD	Full	CD	FSD	Full	CD	FSD	Full	CD	FSD	Full	CD	FSD	Full	CD	FSD
Backdoor	0.98	0.83	0.62	0.82	0.63	0.14	0.11	0.00	0.00	0.26	0.00	0.00	0.89	0.00	0.15	0.97	0.93	0.54
DDoS_HTTP	1.00	1.00	0.91	0.99	1.00	0.94	0.54	0.00	0.01	0.73	0.32	0.17	0.97	1.00	0.00	1.00	1.00	0.94
DDoS_ICMP	1.00	1.00	1.00	1.00	1.00	1.00	1.00	1.00	0.98	1.00	1.00	0.99	1.00	1.00	1.00	1.00	1.00	1.00
DDoS_TCP	1.00	1.00	1.00	1.00	1.00	1.00	1.00	0.94	0.63	1.00	0.96	0.74	1.00	1.00	1.00	1.00	1.00	1.00
DDoS_UDP	0.83	0.83	1.00	1.00	0.17	1.00	1.00	0.17	0.00	0.50	0.00	0.00	1.00	1.00	1.00	1.00	0.83	1.00
Normal	1.00	1.00	1.00	1.00	1.00	1.00	0.99	0.98	0.97	0.99	0.98	0.97	0.67	0.54	0.75	1.00	1.00	1.00
OS_Fingerprinting	0.99	0.97	0.70	0.55	0.15	0.46	0.23	0.00	0.00	0.16	0.00	0.00	1.00	0.08	0.00	1.00	0.97	0.72
Password	0.98	0.94	0.88	0.96	0.88	0.89	0.92	0.67	0.29	0.95	0.81	0.34	0.38	0.78	0.05	1.00	0.98	0.94
Port_Scanning	0.97	0.96	0.82	0.79	0.69	0.75	0.76	0.00	0.01	0.76	0.00	0.12	1.00	0.06	0.36	0.96	0.94	0.82
Ransomware	0.99	0.93	0.74	0.93	0.62	0.41	0.38	0.04	0.00	0.42	0.01	0.00	0.77	0.02	0.39	0.99	0.97	0.79
SQL_injection	1.00	1.00	0.42	0.50	0.63	0.24	0.00	0.00	0.00	0.07	0.00	0.00	0.42	1.00	0.14	1.00	1.00	0.23
Uploading	1.00	0.93	0.66	0.69	0.64	0.56	0.90	0.58	0.00	0.99	0.33	0.02	0.44	1.00	0.10	1.00	0.99	0.58
Vulnerability_scanner	0.97	0.91	0.90	0.83	0.84	0.83	0.83	0.00	0.83	0.83	0.00	0.83	0.83	1.00	0.81	0.91	0.91	0.88
XSS	0.58	0.24	0.17	0.07	0.00	0.10	0.04	0.00	0.00	0.04	0.00	0.00	0.07	0.01	0.00	0.27	0.01	0.15

**Table 11:** Classification performance based on F-measure for the Edge-IIoTset dataset.

Class Label	J48			KNN			LinearSVC			LogisticRegression			NaiveBayes			RandomForest		
	Full	CD	FSD	Full	CD	FSD	Full	CD	FSD	Full	CD	FSD	Full	CD	FSD	Full	CD	FSD
Backdoor	0.98	0.87	0.60	0.88	0.66	0.18	0.20	0.00	0.00	0.40	0.00	0.00	0.09	0.00	0.23	0.98	0.96	0.59
DDoS_HTTP	1.00	1.00	0.89	0.99	1.00	0.88	0.64	0.01	0.02	0.78	0.41	0.26	0.98	1.00	0.00	1.00	1.00	0.89
DDoS_ICMP	1.00	1.00	1.00	1.00	0.99	1.00	0.99	0.99	0.97	0.99	0.99	0.98	1.00	1.00	0.99	1.00	1.00	1.00
DDoS_TCP	1.00	1.00	1.00	1.00	0.99	1.00	1.00	0.91	0.71	1.00	0.92	0.79	0.98	0.97	0.71	1.00	1.00	1.00
DDoS_UDP	0.91	0.91	0.67	1.00	0.29	0.67	0.75	0.29	0.00	0.67	0.00	0.00	1.00	1.00	0.67	1.00	0.91	0.67
Normal	1.00	1.00	1.00	1.00	1.00	1.00	0.99	0.95	0.88	0.99	0.97	0.90	0.80	0.70	0.82	1.00	1.00	1.00
OS_Fingerprinting	0.99	0.97	0.76	0.66	0.25	0.52	0.37	0.00	0.00	0.27	0.00	0.00	0.77	0.14	0.00	1.00	0.94	0.77
Password	0.98	0.95	0.88	0.92	0.88	0.86	0.81	0.59	0.39	0.86	0.67	0.44	0.41	0.27	0.09	0.98	0.97	0.90
Port_Scanning	0.97	0.95	0.82	0.86	0.78	0.83	0.80	0.00	0.03	0.84	0.00	0.21	0.17	0.10	0.11	0.96	0.96	0.83
Ransomware	0.99	0.93	0.74	0.91	0.67	0.48	0.48	0.07	0.00	0.50	0.03	0.00	0.07	0.04	0.05	0.99	0.96	0.76
SQL_injection	1.00	1.00	0.44	0.61	0.70	0.30	0.00	0.00	0.00	0.09	0.00	0.00	0.55	0.76	0.18	1.00	1.00	0.32
Uploading	1.00	0.92	0.65	0.79	0.72	0.62	0.89	0.68	0.00	0.90	0.47	0.03	0.60	0.88	0.16	1.00	0.98	0.66
Vulnerability_scanner	0.97	0.90	0.91	0.88	0.77	0.88	0.88	0.00	0.87	0.88	0.00	0.88	0.88	0.55	0.80	0.95	0.92	0.93
XSS	0.57	0.23	0.17	0.13	0.00	0.18	0.08	0.00	0.00	0.07	0.00	0.00	0.13	0.01	0.00	0.41	0.02	0.24

**Table 12:** Summary of classification accuracy.

Dataset	J48		KNN		LinearSVC		Logistic Regression		NaiveBayes		RandomForest	
	CD	FSD	CD	FSD	CD	FSD	CD	FSD	CD	FSD	CD	FSD
N-BaIoT	8 / 9	9 / 9	6 / 9	9 / 9	1 / 9	1 / 9	2 / 9	3 / 9	6 / 9	6 / 9	8 / 9	9 / 9
CICIoT2023	7 / 8	2 / 8	4 / 8	5 / 8	3 / 8	3 / 8	3 / 8	3 / 8	3 / 8	3 / 8	7 / 8	2 / 8
Edge-IIoTset	12 / 14	4 / 14	8 / 14	7 / 14	3 / 14	2 / 14	3 / 14	3 / 14	6 / 14	4 / 14	13 / 14	5 / 14

**Table 13:** Influence of (Full/CD/FSD) on the mean training time (ms) of classifiers on different datasets.

Classifier	N-BaIoT			CICIoT2023			Edge-IIoTset		
	Full	CD	FSD	Full	CD	FSD	Full	CD	FSD
LinearSVC	17,073	63	2325	107,377	449	23,941	120,607	269	151,133
NaiveBayes	179	10	41	195	4	78	470	17	158
J48	5422	133	2919	2498	74	2941	2574	53	1357
RandomForest	56,620	3247	47,878	35,287	1874	80,371	33,025	1376	43,326
KNN	1759	15	184	2122	21	590	5485	40	954
LogisticRegression	65,510	658	23,070	73,650	1011	46,532	394,870	4219	171,205

Speed-up ratio—averaging times across all classifiers and datasets for each approach: Full / CD indicates that CD is 68× faster than Full (overall). Full / FSD indicates that FSD is 1.5× faster than Full (overall). FSD / CD indicates that CD is 45× faster than FSD (overall).

**Table 14:** Influence of (Full/CD/FSD) on the mean testing time (ms) of classifiers on different datasets.

Classifier	N-BaIoT			CICIoT2023			Edge-IIoTset		
	Full	CD	FSD	Full	CD	FSD	Full	CD	FSD
LinearSVC	20	14	18	26	19	16	46	32	43
NaiveBayes	396	48	53	347	142	120	1368	362	427
J48	15	6	19	30	6	29	47	33	32
RandomForest	500	446	542	1392	1123	3049	2350	2100	3909
KNN	207,015	1938	8999	2,204,285	10,745	331,032	10,658,362	26,787	295,290
LogisticRegression	22	6	22	16	16	16	48	25	52

Speed-up ratio—averaging times across all classifiers and datasets for each approach: Full / CD indicates that CD is 298× faster than Full (overall). Full / FSD indicates that FSD is 20× faster than Full (overall). FSD / CD indicates that CD is 15× faster than FSD (overall).

**Table 15:** Influence of (Full/CD/FSD) on the RAM usage (MB) of classifiers across different datasets.

Classifier	N-BaIoT			CICIoT2023			Edge-IIoTset		
	Full	CD	FSD	Full	CD	FSD	Full	CD	FSD
J48	2.88	0.0859	2.43	2.46	0.0039	5.88	1.15	0.3477	4.80
KNN	140.60	1.15	20.21	135.11	3.02	47.58	307.16	5.05	72.89
LinearSVC	5.61	0.0938	6.20	12.55	0.6836	0.0508	7.32	0.2109	4.64
LogisticRegression	130.68	2.08	33.77	128.90	2.79	78.67	288.55	4.12	107.91
NaiveBayes	1.19	0.2188	0.7383	1.14	0.1523	0.0508	7.76	2.47	0.2383
RandomForest	5.97	0.3320	0.2148	146.45	8.27	488.91	102.88	18.41	7.38

Averaging RAM usage across all classifiers and datasets for each approach: CD has the smallest memory footprint overall— 29× less than Full and 18× less than FSD. FSD uses 1.6× less memory than Full overall.

## Data Availability

The data are publicly available, and each dataset is cited with its repository in the paper.
